# A serosurvey for bovine respirovirus 3 in Turkish domestic ruminants: The first comparison study of A and C genotypes

**DOI:** 10.1002/vms3.534

**Published:** 2021-05-25

**Authors:** Bahadir Muftuoglu, Hanne Nur Kurucay, Ahmed Eisa Elhag, Serdar Yildirim, Yasemin Cicek‐Yildiz, Cuneyt Tamer, Emre Ozan, Kezban Can Sahna, Yakup Yildirim, Harun Albayrak, Semra Okur‐Gumusova, Zafer Yazici

**Affiliations:** ^1^ Department of Veterinary Experimental Animals Faculty of Veterinary Medicine Ondokuz Mayis University Samsun Turkey; ^2^ Department of Veterinary Virology Faculty of Veterinary Medicine Ondokuz Mayis University Samsun Turkey; ^3^ Department of Preventive Medicine and Clinical Studies Faculty of Veterinary Sciences University of Gadarif Al Qadarif Sudan; ^4^ Ministry of Agriculture and Forestry Samsun Veterinary Control Institute Samsun Turkey; ^5^ Department of Virology Faculty of Veterinary Medicine University of Firat Elazig Turkey; ^6^ Department of Virology Faculty of Veterinary Medicine Burdur Mehmet Akif Ersoy University Burdur Turkey

**Keywords:** BPIV‐3, genotype A, genotype C, neutralised antibodies, ruminants

## Abstract

Bovine parainfluenza virus‐3 (BPIV‐3), also known as bovine respirovirus 3, causes serious respiratory infection in ungulates, often involving other pathogens, such as viruses, bacteria and mycoplasmas. In this study, we evaluated antibody titers against virus genotypes A (BPIV‐3a) and C (BPIV‐3c). We conducted a serological survey and comparison analysis of archived serum samples from small and large ruminants reared in four Turkish provinces. A total of 1,307 samples, consisting of sheep (*n* = 444), cattle (*n* = 402), water buffalo (*n* = 261) and goat (*n* = 200) sera, were randomly selected from stock samples collected between 2015 and 2019 and screened by standard virus neutralisation assay. We found that 49.9% (653/1307) of all samples were positive for neutralising antibody titers. Goats had the highest titer, with total seropositivity of 63% (126/200), followed in descending order by cattle, sheep and water buffalo at 56.2% (226/402), 32.2% (143/444) and 26% (68/261) total seropositivity, respectively. BPIV‐3c had the highest neutralising antibody rate at 34.3% (448/1307), whereas BPIV‐3a had a 24.3% (317/1307) seropositivity rate. Neutralising antibody titers for positive samples ranged between 1/4 and 1/512 per the SN_50_ test. Seropositivity rates ranged from a low of 8.9% to a high of 18.3%. Our study was the first to compare antibody seroprevalence for two BPIV‐3 genotypes in small and large domestic ruminants, which were shown to be more commonly exposed to BPIV‐3c than BPIV‐3a. This finding could have significant implications as current vaccines mainly use the BPIV‐3a genotype. Further research can determine if current vaccines protect against different BPIV‐3 virus genotypes.

## Graphical abstract

Our study was the first attempt to compare antibodies prevalence for two genotypes (A and C) of bovine respirovirus 3 in small and large domestic ruminants that were proved to be exposed more commonly to BPIV‐3c than BPIV‐3a, which may indicate that A may not be the dominant genotype of BPIV‐3. This may influence Turkish livestock production and offer important implications for animal health. Due to the critical role, this virus plays in BRDC pathogenesis and the risk that arises from using current vaccines which mainly uses the BPIV‐3a genotype.

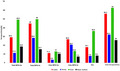

## 1 INTRODUCTION

Bovine parainfluenza virus‐3 (BPIV‐3), also known as bovine respirovirus 3, is a non‐segmented, single‐stranded, negative‐sense and enveloped RNA virus. It is a member of the *Respirovirus* genus and classified under the sub‐family *Orthoparamyxovirinae* of the family *Paramyxovirida*e, under the order *Mononegavirales* (ICTV, 2019; King et al., 2012). This virus causes serious respiratory infections in ungulates and may cause illness alone or in mixed infections with other pathogens, mainly viruses, bacteria and mycoplasmas. BPIV‐3, as part of this mixed infection, manifests when the stresses of long‐distance transport weaken an animal's immune system and increase its vulnerability to infection. This may result in the disease popularly known as ‘shipping fever’ or bovine respiratory disease complex (BRDC) (Elankumaran, 2013). BPIV‐3 is a contributor to BRDC, a global cattle health problem that causes significant economic losses in stocker and feedlot production systems (Edwards, 2010). Other viruses contributing to the BRDC are bovine respiratory syncytial virus (BRSV), bovine viral diarrhoea virus (BVDV), bovine adenovirus type 3 (BAV‐3) and bovine herpes virus‐1 (BHV‐1), along with other bacterial species including *Mannheimia/Pasteurella*, *Haemophilus/Histophilus* and *Mycoplasma* spp. (Fulton, 2009). BRDC severity can be exacerbated by increased susceptibility to secondary bacterial infection with one or all the aforementioned species, along with possible risk factors, such as transportation, hygiene, co‐mingling, stocking density, host immune status and environmental temperature (Gagea et al., 2006; Snowder et al., 2006). However, recent studies have reported both severe and fatal BRDC cases in cattle linked to individual pathogens, such as BPIV‐3, BHV‐1 or BRSV (Albayrak et al., ,^2019^, ^2020^; Yazici et al., 2020).

The BPIV‐3 virus was first identified in the United States in 1959, when virus was isolated from nasal swabs of calves with symptoms including lack of appetite, coughing, nasal discharges, other respiratory signs, fever, lacrimation and conjunctivitis (Gueriche et al., 2020). BPIV‐3 has since been reported worldwide in both asymptomatic and clinically affected cattle and high prevalence of BPIV‐3‐specific antibodies has been noted, especially in beef and dairy herds. The virus is endemic in parts of Asia, Europe, North and South America (Spilki, 2016). Although BPIV‐3 is usually found in cattle and small ruminants, infections have been reported in buffalo, camelids, horses, pigs, dogs and monkeys as well as cross‐species infections in humans (Giangaspero et al., 2013; Intisar et al., 2010; Maidana et al., 2012; Yener et al., 2005). Although the virus shares roughly 25% cross‐neutralisation and has genetic and antigenic similarities with human parainfluenza virus type‐3 (HPIV‐3), human to human transmission is very rare. However, animal to animal transmission can occur by indirect and direct contact through aerosols and fomites contaminated with nasal discharges. The resulting disease generally remains subclinical in calves, lambs and kids but may present as pneumonia and acute respiratory infection (Skiadopoulos et al., 2003) characterised by low morbidity and rare mortality (Theurer et al., 2015). Morbidity and mortality can be higher in cases of co‐infection with other viral or bacterial pathogens (Fulton, 2009). BPIV‐3 mostly affects cattle aged two to six months, likely following the animal's declining maternal‐derived passive immunity, although several outbreaks have been reported in younger animals (Ellis, 2010). Moreover, additional stresses resulting from harsher climates in many countries, along with accumulating treatment costs, lower growth performance, declining carcass value and high mortality rates, contribute to significant losses for dairy and beef farms of approximately 1 billion US dollars per year (Griffin, 1997).

Since the 1960s, many vaccines have been developed for BPIV‐3, mainly live attenuated and inactivated types administered via parenteral and intranasal routes. Currently available vaccines are mainly combination‐type, formulated with various viral and bacterial antigens that constitute BRDC. However, there is a controversy and alarm over their use in the field due to concerns that they contain other pathogens related to BRDC. Other concerns also include potential factors such as maternal immunity and environmental conditions (Theurer et al., 2015).

The BPIV‐3 virions are pleomorphic and have a diameter ranging from 150 to 250 nm. Genome length is more than 15,000 nt and comprises six genes encoding nine proteins. Among these nine proteins, the Hemagglutinin‐Neuraminidase (HN) and fusion envelope proteins are responsible for viral attachment and penetration (Newcomer et al., 2017; Zhu et al., 2011). Although there is an antigenic similarity among BPIV‐3 isolates, the nucleotide level reveals viral diversity; three distinct genotypes (A, B and C) that are difficult to clinically differentiate have been described, and subgenotypes may occur (Ellis, 2010). Previous complete genome analyses of BPIV‐3 representative isolates indicated that genotype A (BPIV‐3a) is distributed geographically worldwide, whereas genotype B (BPIV‐3b) is located primarily in Australia (Horwood et al., 2008). A new genotype was later proposed as C (BPIV‐3c) and detected from isolates in Argentina, Korea and China (Maidana et al., 2012; Oem et al., 2013; Zhu et al., 2011).

BPIV‐3 antibodies are thought to be prevalent in almost 80% of dairy and beef cattle, which may demonstrate broad virus dissemination (Figueroa‐Chávez et al., 2012). Data on virologic detection of this virus in Turkey are limited, but serological detection is widely reported, and few molecular cases are reported (Albayrak et al., 2019; Alkan et al., 2000; Timurkan et al., 2019). Turkish seroprevalence distribution in local ruminant herds is ranged from low (11%) (Timurkan et al., 2019) to high (92.8%) (Duman et al., 2009). To our knowledge, most serological surveys were conducted in selected regions (Yavru et al., 2005; Yesilbag & Gungur, 2009) or countrywide (Alkan et al., 2000) and used genotype A of the virus. The exception is a recent study in which the first Turkish indigenous genotype C isolate was used, in which the overall seropositivity rate was 21.1% (Yazici et al., 2019). The aim of this study was to conduct field surveillance to measure possible variations between A and C genotypes of BPIV‐3. We sought to make a serological survey and a comparison analysis for the detection of antibodies against both A and C genotypes of the parainfluenza‐3 virus (BPIV‐3) in small and large ruminants reared in different Turkish provinces.

## 2 MATERIALS AND METHODS

### 2.1 Sampling

**FIGURE 1:**
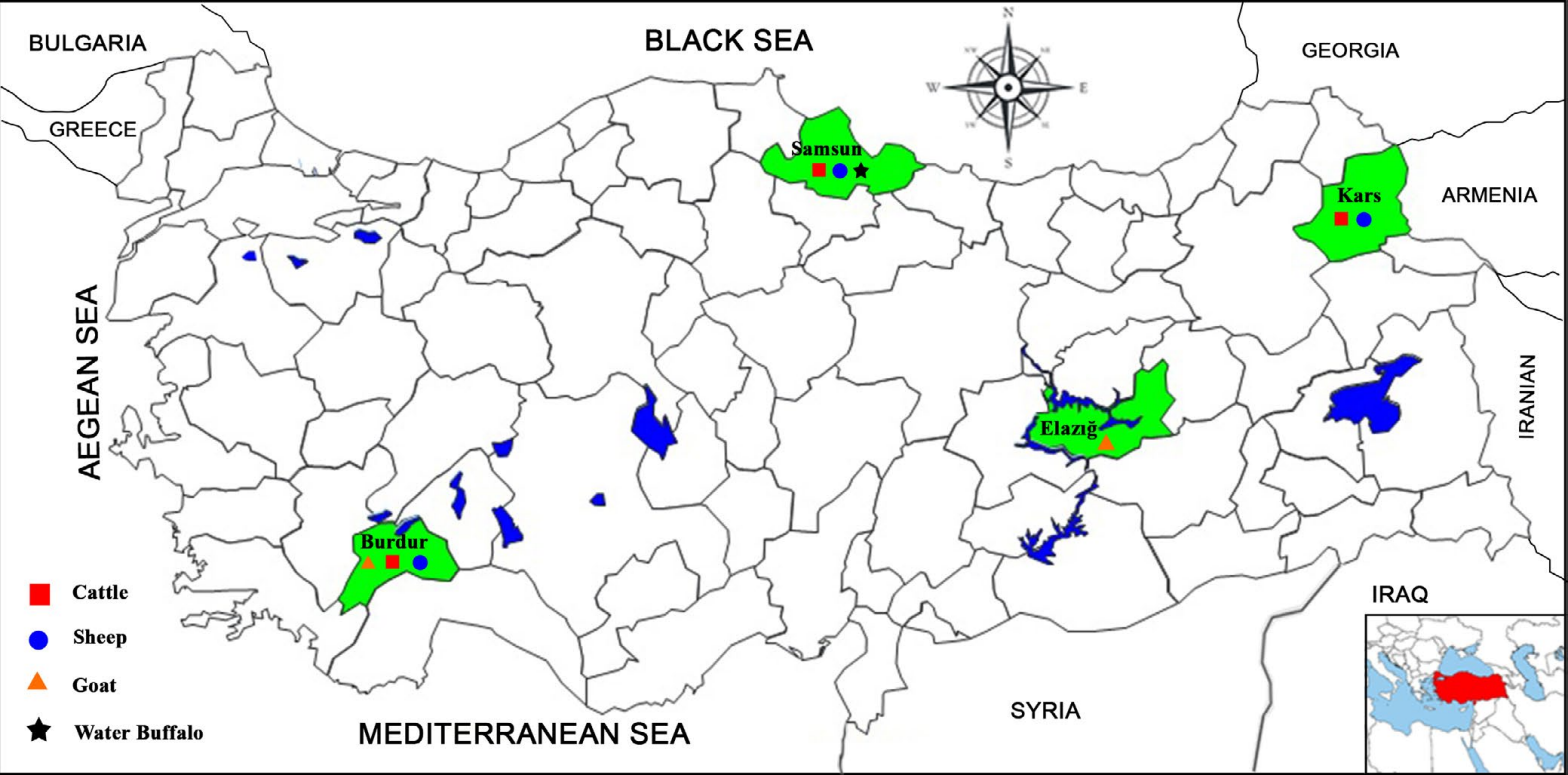
Map indicates the four different provinces in Turkey where cattle (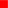), sheep (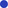), goat (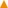) and water buffalo (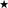) serum samples were collected

A total of 1,307 archived serum samples consisting of sheep (*n* = 444), cattle (*n* = 402), water buffalo (*n* = 261) and goats (*n* = 200) sera were randomly selected from stock samples collected from four provinces in different Anatolian regions between 2015 and 2019. Cattle and sheep samples came from three provinces (Burdur, Kars and Samsun), goat samples from two (Burdur and Elazığ) and water buffalo samples from one (Samsun) (Figure 1). Sampled animals were 1 year or older and none were vaccinated against the viral pathogens of BRDC. The serum samples were heat‐inactivated for 30 min at 56°C before serum neutralisation assay occurred.

### 2.2 Viruses and cell culture

For this study, we obtained the required viruses and cell culture lines from our Virology Department. The SF4 German reference strain of BPIV‐3a and the local Turkish BPIV‐3c isolate (Albayrak et al., 2019) with the Genbank accession no: MH357343 were propagated in culture flasks containing MDBK cells as described previously (Yazici et al., 2019). Culture virus‐containing supernatant was harvested after a visible cytopathic effect (CPE) was observed, and the monolayer was destroyed. The collected supernatant was then clarified in a centrifuge at 3,000 rpm for 5 min, then frozen at −80°C until further use.

Before commencing the study, all cell lines and cell culture reagents were checked for non‐cytopathogenic (NCP) pestiviruses contamination by using a real‐time RT‐PCR method as previously described (Yazici et al., 2017). Madin Darby Bovine Kidney (MDBK) cells grown in Dulbecco's modified Eagle medium (DMEM, Gibco, UK), supplemented with 1% antibiotic solutions (25,000 units of penicillin and streptomycin) (Sigma‐Aldrich, St. Louis, MO, USA) and 10% fetal bovine serum (FBS, Gibco, Paisley, UK) were used to perform virus cultivation, neutralisation and infectivity assays.

### 2.3 Infectivity assay

The two viruses were titrated according to previously mentioned protocols (Yazici et al., 2015). They were diluted 10‐fold in DMEM with supplements of 2% FBS. Ninety‐six well plates (TPP, Trasadingen, Switzerland) were used for this assay, and 100 μl of each dilution was put in every well into quadruplicates. Fifty microlitre of MDBK cells were then added to each well, each containing a 3.0 × 104 cell suspension. The plates were placed in a humidified incubator (at 5% CO_2_) for 72 hr at 37°C. A 50% tissue culture infective dose (TCID_50_) was calculated as TCID_50_/ml following incubation.

### 2.4 Neutralisation assays

To detect neutralising antibodies against both BPIV‐3a and BPIV‐3c, a standard virus neutralisation assay was used as described before (Yazici et al., 2015). Half (1/2) dilutions of all serum samples were made in 96‐well plates using 50 μl of each serum diluted in DMEM containing 2% FBS. Then 100 TCID_50_ of the two viruses were added to each well. The plates were placed in a humidified incubator (at 5% CO_2_) for 1 hr at 37°C. Finally, 50 μl of a cell suspension containing 3.0 × 10^4^ MDBK was added to each well, followed by incubation (at 5% CO_2_) at 37°C for 72 hr. Protection was evaluated according to the presence of CPE. Any antibody titer detected as <1/2 from serum samples was considered to be seronegative. A 50% serum neutralisation test (SN_50_) was performed to determine the antibody titers of positive sera. Two‐fold dilutions of all positive samples were prepared across a range of 1/4–1/512 using an otherwise identical procedure to that described above.

### 2.5 Statistical analysis

An R Studio statistical analysis programme was used to analyse the data. A χ^2^ test was also used to compare the group's differences. We compared animal species according to seropositivity rates for A and C genotypes of BPIV‐3 and the results were considered to be statistically significant at *p* < .05.

## 3 RESULTS

**FIGURE 2:**
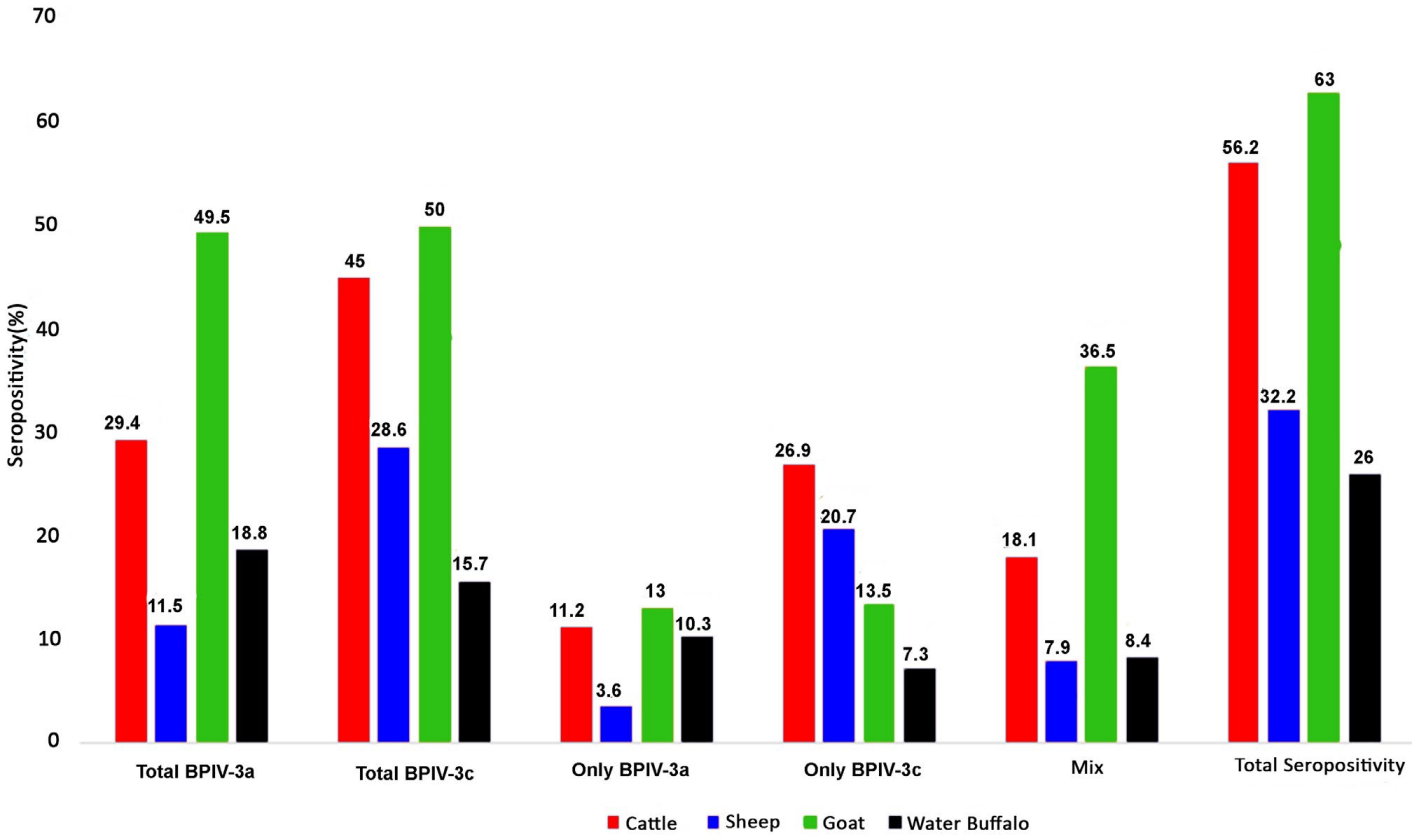
Distribution of BPIV‐3 seropositivity in various ruminant species according to animal species and virus genotypes

The overall seropositivity was 49.9% (663/1307), with 24.3% (317/1307) for genotype A and 34.3% (448/1307) for genotype C. Species‐specific seropositivity was 63% (126/200), 56.2% (226/402), 32.2% (143/444) and 26% (68/261) for goats, cattle, sheep and water buffalo, respectively (Table 1 and Figure 2). The province of Elazığ had the highest overall BPIV‐3 seropositivity with 67.3%, followed by Burdur at 59.3%, Kars at 53% and Samsun at 45.2% in descending order. (Table 1).

**TABLE 1:** Distribution of BPIV‐3 seropositivity in various ruminant species according to province, animal species and virus genotypes

Provinces	Number and species of sampled animals					BPIV−3 seropositivity (%)					
	Cattle	Sheep	Goat	Water buffalo	Total	Only		Mix	Total		Overall seropositivity
						BPIV−3a	BPIV−3c		BPIV−3a	BPIV−3c	
Burdur	50	50	50	—	150*	2.7 (4/150)	18.7 (28/150)	38 (57/150)	40.7 (61/150)	56.7 (85/150)	**59**.**3** **(89/150)**
Elazığ	—	—	150	—	150*	17.3 (26/150)	12.7 (19/150)	37.3 (56/150)	54 (81/150)	49.3 (74/150)	**67.3** **(101/150)**
Kars	50	50	—	—	100*	14 (14/100)	8 (8/100)	31 (31/100)	45 (45/100)	39 (39/100)	**53** **(53/100)**
Samsun	302	344	—	261	907*	7.7 (70/907)	30.9 (280/907)	6.6 (60/907)	14.3 (130/907)	27.6 (250/907)	**45.2** **(410/907)**
**Total**	**402**	**444**	**200**	**261**	1,307**	**8.7 (114/1307)**	**25.6 (335/1307)**	**15.6** **(204/1307)**	**24.3 (317/1307)**	**34.3 (448/1307)**	**49.9 (653/1307)**

Bold indicates the overall and total results.

* There is a significant statistical difference among all provinces with a total *p*‐value of < 0.05

** Significant statistical differences were determined between all animal species with a total *p*‐value of < 0.05.

A detailed serum neutralisation test using both genotypes A and C of BPIV‐3 is shown in Table 1 revealed that BPIV‐3c has the highest neutralising antibody rate in all tested samples, at 34.3% (448/1307) compared with BPIV‐3a at 24.3% (317/1307) seropositivity rate. We also obtained results regarding the co‐infection of both genotypes in the same animals, recording a mixed infection seroprevalence of 15.6% (204/1307) for both BPIV‐3a and BPIV‐3c in all tested samples. If we considered this when calculating the unmixed seroprevalence for both BPIV‐3a and BPIV‐3c, it would be 8.7% (114/1307) and 25.6% (335/1307), respectively.

As detailed in Figure 2, goats had the highest rate of total seropositivity at 63%, followed by cattle, sheep and water buffalo, which showed 56.2%, 32.2% and 26% seroprevalence, respectively. The highest seropositivity rate for total BPIV‐3a was found in goats at 49.5% followed by cattle at 29.4%, water buffalo at 18.8% and sheep at 11.5%. Additionally, the highest seropositivity for total BPIV‐3c was found in goats at 50%, followed by cattle at 45%, sheep at 28.6% and water buffalo at 15.7%. When determining the co‐infection of both A and C genotypes of BPIV‐3, we found that goats had the highest rate at 36.5%, followed by cattle at 18.1%, water buffalo at 8.4% and sheep at 7.9%. When we calculated only BPIV‐3a seroprevalence without co‐infection rates, goats had the highest seropositivity rate at 13%, followed by cattle, water buffalo and sheep at 11.2%, 10.3% and 3.6% seroprevalence, respectively. The situation was different when we calculated BPIV‐3c seroprevalence without co‐infection rates, as cattle showed a 26.9% seropositivity rate, considered the highest among ruminant species, followed in descending order by sheep at 20.7%, goats at 13.5% and water buffalo at 7.3% (Figure 2).

**FIGURE 3:**
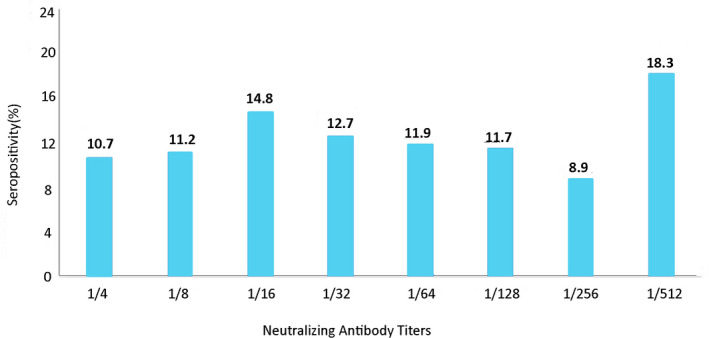
The distribution of BPIV‐3 total neutralising antibody titers in all ruminant species

As detailed in Figure 3, neutralising antibody titer distribution in positive samples ranged between 1/4 and 1/512 antibody titers per SN_50_ testing. Seropositivity rates ranged between 8.9% and 18.3%.

## 4 DISCUSSION

In the current study, neutralising antibody titers for BPIV‐3c recorded the highest rate among tested samples at 34.3%, whereas BPIV‐3a showed a 24.3% seropositivity rate. This shows the increased emergence of BPIV‐3 non‐A genotypes. Potential reasons for these findings may include geographic isolation and a dynamic shift of the virus population due to the wide use of commercial vaccines including only BPIV‐3a. Such use may allow non‐A genotypes an opportunity to emerge, especially when we know that minimum cross‐protection is achieved within antibodies against different genotypes of BPIV‐3 (Newcomer et al., 2017). Most previous reports proposed that genotypes B and C were found in a few countries, like the United States, Australia, Argentina, Korea and China (Maidana et al., 2012; Oem et al., 2013; Zhu et al., 2011). However, this assumption is changing as both have begun spreading globally (Newcomer et al., 2017; Wen et al., 2017). A scenario envisioning the rising prevalence of emergent genotype C in Turkish local domestic ruminant herds could be drawn, especially when considering Turkey's strategic location at the crossroads of Eurasian global trade. This position may enable a spillover of genotype C to Turkey's ungulates, in light of the country's extensive livestock importation business with over 15 countries from different continents, particularly with Uruguay, Brazil and Argentina (Okur‐Gumusova et al., 2020). However, future studies on virus isolation and sequencing are needed to gain a clear picture of the origins of genotype C of the BPIV‐3.

To the best of our knowledge, our study was the first to compare antibody seroprevalence for the two genotypes (A and C) of BPIV‐3 in small and large unvaccinated domestic ruminants (goats, sheep, cattle and water buffalo), with findings indicating the animals are more commonly exposed to BPIV‐3c than BPIV‐3a; our study was similar to previous molecular characterisation investigation which indicated that half of the obtained isolates belonged to BPIV‐3c and less than 25% of isolates were classified as BPIV‐3a (Fulton et al., 2017). In contrast, the only serological study comparing all three genotypes (A, B and C) of BPIV‐3 in unvaccinated ungulates in the United States found that BPIV‐3b was the most frequently seen genotype compared with the others (Newcomer et al., 2017). Nevertheless, our results revealed that field infections with BPIV‐3c are increasing in comparison with BPIV‐3a, raising the possibility that it may no longer be the main genotype of the virus in northern, eastern, central and southwestern Turkey and possibly globally. Further wide surveillance studies in the provinces, along with investigating other genotypes like BPIV‐3b (not employed in our study), would explore and confirm the possibility of national change in the type of genotypes prevalent in BPIV‐3.

Worldwide prevalence of BPIV‐3a is relatively high, but along with geographic isolation, the use of commercial kits for virus diagnosis could play a role in this predominance (Yazici et al., 2019). These kits are mainly genotype A antisera and antigen inclusive and may raise questions about their limitations in diagnosing other BPIV‐3 genotypes (Neil et al., 2015). Our findings indicated that genotype A may no longer be the dominant BPIV‐3 genotype circulating among ruminants in Turkey. Such results could significantly affect the vaccines currently used to protect dairy and beef cattle from BRDC pathogens on Turkish farms. Although these vaccines are genotype A inclusive, it is unknown if they are protective agianst genotype C of BPIV‐3. As the ruminants in our study were not vaccinated against the pathogens that constitute BRDC and were past the age of having active and persistent maternal antibodies (Ellis, 2010), the antibody titers we obtained from seropositive ruminants seem to be a consequent of natural exposure to respective strains of BPIV‐3. Although the antibodies generated against BPIV‐3 are higher in prevalence in older animals, the titers and duration of antibodies required for disease protection is unknown, as most field cases are passing undetected (Newcomer et al., 2017; Spilki, 2016).

In the present study, the overall neutralising antibody positive rate for BPIV‐3 virus detected from all samples was 49.9%, while total seropositivity rates for goats, cattle, sheep and water buffalo were 63%, 56.2%, 32.2% and 26%, respectively. In various serological reports obtained from local ruminant herds in Turkey, the total neutralising antibody positive rate for BPIV‐3 ranged from low (21.1%) to high (88.8%) (Alkan et al., 2000; Okur‐Gumusova et al., 2007; Yazici et al., 2007, 2019; Yesilbag˘ & Gungor, 2009). Surprisingly our results showed that co‐infection with both genotypes can be seen in the same animal. For example, we recorded a total mixed infection seroprevalence of 15.6% for both BPIV‐3a and BPIV3‐c in all tested samples. This clearly indicates that BPIV‐3 infection rates are increasing as a result of rising mixed infections. On the other hand, our study recorded the unmixed (single) seroprevalence for BPIV‐3a and BPIV‐3c at 8.7% and 25.6%, respectively. Previous studies of single seroprevalence rates for BPIV‐3a in Turkish ruminants estimated a range of 0.2%–6.5%, slightly lower than our results (Alkan et al., 2000; Okur‐Gumusova et al., 2007; Yesilbag & Gungor, 2009). Moreover, no single seroprevalence rates for BPIV‐3c were detected in Turkey, except from a new study that showed a prevalence of 21.1% (Yazici et al., 2019), a figure slightly lower than our single BPIV‐3c prevalence. As long as there is no benchmark of antibody cross‐reaction to different genotypes of BPIV‐3, it is unknown if our sampled ruminants gained both A and C virus genotypes through cross‐reaction or natural exposure, acknowledging the fact that some antigenic differences are reported within genotypes and subgenotypes of BPIV‐3 (Neil et al., 2015). Consequently, future studies are needed to clarify the degree of cross‐reaction within antibodies towards all three genotypes of BPIV‐3 and to identify any peculiar serotypes that may appear.

Furthermore, although cattle are the main species associated with BPIV‐3, there are reports of cross‐transmission between cattle and goats, as well as sheep and water buffalo (Maidana et al., 2012; Yesilbag & Gungor, 2009). Our findings show evidence for cross‐species transmission by indicating a 63% seropositivity for goats, much higher than those we detected in cattle, sheep and water buffalo and higher than the previously reported 19.7% rate of seroprevalence in goats from Turkey (Yesilbag & Gungor, 2009). We also detected a 32.2% seroprevalence rate for sheep, much higher than the previous rate of approximately 8.8% from local sheep herds (Yesilbag & Gungor, 2009). These findings may suggest that small ruminants, especially goats with high BPIV‐3 prevalence, can act as a reservoir or a carrier in the transmission of BRDC to cattle, particularly when the main cause involves viruses like BPIV‐3, given the fact that our sampled animals were herded together in various province locations. This is also relevant for genotypes A and C of BPIV‐3, as their highest seropositivity was detected in goats, with lower rates found in other species. This indicates that cross‐transmission for BPIV‐3 can be achieved between species in both A and C virus genotypes and that reservoir animals can carry more than one virus genotype.

## 5 CONCLUSION

In this study of large and small ruminants of Turkey, BPIV‐3c antibody titers were found to be significantly higher than BPIV‐3a titers. These results indicate that non‐A genotypes of the virus, especially BPIV‐3c, could eventually replace BPIV‐3a as the predominate genotype circulating among local Turkish herds. Despite the lack of information on antibody cross‐reaction against different genotypes of BPIV‐3, the dynamic shift of virus genotypes detected in field strains may influence Turkish livestock production and offer important implications for animal health. This is due to the critical role this virus plays in BRDC pathogenesis and the advantage it may have due to the current use of BPIV‐3a vaccines that may not address other genotypes. Thus, future research is needed to define the level of protection that current vaccines offer for different BPIV‐3 genotypes.

## CONFLICT OF INTEREST

No conflict of interest is declared for this study.

## AUTHORS CONTRIBUTION

**Bahadır Muftuoglu:** Formal analysis; Methodology; Software; Writing‐review & editing. **Hanne Nur Kurucay:** Formal analysis; Methodology; Visualization. **Ahmed Eisa Elhag:** Formal analysis; Methodology; Writing‐original draft; Writing‐review & editing. **Serdar Yildirim:** Investigation; Resources. **Yasemin Cicek‐Yildiz:** Investigation; Resources. **Cuneyt Tamer:** Formal analysis; Writing‐review & editing. **Emre Ozan:** Validation; Writing‐review & editing. **Kezban Can Sahna:** Conceptualization; Resources. **Yakup Yildirim:** Conceptualization; Resources. **Harun Albayrak:** Conceptualization; Supervision. **Semra OKUR GÜMÜŞOVA:** Conceptualization; Supervision. **Zafer Yazici:** Conceptualization; Supervision; Visualization; Writing‐review & editing.

## ETHICS STATEMENT

The authors confirm that the ethical policies of the journal, as noted on the journal's author guidelines page, have been adhered to. No ethical approval was required, as no animals were culled or treated during this study. We obtained permission for this work from the Experimental Animals and Local Ethics Committee, Samsun Veterinary Control Institute, Ministry of Food, Agriculture and Livestock, Turkey (Approval No. 2/2020 dated 28 February 2020).

### PEER REVIEW

The peer review history for this article is available at https://publons.com/publon/10.1002/vms3.534.
